# Salvarsan and Parasyphilis

**Published:** 1915-06

**Authors:** 


**Salvarsan and Parasyphilis.** Schwarz, Deutsch. Zeit. fuer Nervenheilkunde, No. 3 and 4, Vol. 52, states that paresis is not curable, though it may be improved for a considerable period. Tabes is curable.

